# Draft genome of *Chryseobacterium cupriresistens* Str01 isolated from a vegetable countertop in a public market in the Philippines

**DOI:** 10.1128/mra.00519-26

**Published:** 2026-07-10

**Authors:** Geraldine B. Dayrit, Kathyleen S. Nogrado, Arienne Joyce O. Amodia, Daniel G. Dalisay, Karen Angelica R. Domalaon, Cayel Jurist C. Garong, Renz Amador I. Bagas, Fresthel Monica M. Climacosa

**Affiliations:** 1 Department of Medical Microbiology, College of Public Health, University of the Philippines Manila 54725, Manila, Philippines; 2 One ARM Program, Department of Medical Microbiology, College of Public Health, University of the Philippines Manila 54725, Manila, Philippines; 3 Institute of Biology, University of the Philippines Diliman 172582, Quezon City, Philippines; 4 Biomedical Innovations Research for Translational Health Sciences, College of Medicine, University of the Philippines Manila, Manila, Philippines; University of Wisconsin-Madison, Madison, Wisconsin, USA

**Keywords:** antibiotic resistance genes, *Chryseobacterium*, *Chryseobacterium cupriresistens*, *Chryseobacterium indologenes*, heavy-metal resistance genes, multidrug-resistance

## Abstract

This announcement presents the genome of *Chryseobacterium cupriresistens* Str01, initially misidentified as *Chryseobacterium indologenes* by VITEK 2 and BLASTn, isolated from a countertop of a vegetable market in Manila, Philippines. Its ~5 Mbp (36.41% G + C) genome carries predicted antibiotic and heavy-metal resistance genes, the latter suggesting adaptive potential in metal-contaminated environments.

## ANNOUNCEMENT

A vegetable countertop from a public market in Manila, Philippines (14°36′19.786″ N, 120°57′50.006″ E) was sampled using Copan ESwab (Copan, USA) in November 2024 as part of an antimicrobial resistance study. The transport solutions were serially diluted on nutrient broth, plated on sheep’s blood agar, and incubated aerobically at 37°C for 24 h. Morphologically distinct bacterial isolates were recovered and subcultured in nutrient agar under static conditions for microbial identification and antibiogram profiling using VITEK 2 AST-N261 (v9.03.3; bioMerieux, France) in Philippine General Hospital (Manila, Philippines). A colony exhibiting a characteristic orange pigment was identified as *Chryseobacterium indologenes*, an emerging public health concern exhibiting multidrug resistance to clinically relevant antibiotics (1–3). Due to limited genomic data for *C. indologenes* in the Philippines, whole genome sequencing (WGS) was performed.

Genomic DNA was extracted using the DNeasy Blood and Tissue Kit (QIAGEN, Germany), quantified using the DS-11+ spectrophotometer (DeNovix, USA), concentrated with a centrifugal evaporator without shearing or size selection, and sent to Adaptor Genomic Sciences LTD. (Kaohsiung, Taiwan) for WGS. Libraries were prepared using the KAPA HyperPrep Kit (Roche, USA) and the Native Barcoding kit (Oxford Nanopore Technologies, UK) and sequenced on PromethION R10.4.1 flow cell using an ONT P2 Solo sequencer. Data acquisition was performed using MinKNOW (v24.06.16), while demultiplexing, trimming, and filtering (*Q* ≥ 7) were carried out using Dorado (v7.4.14, high-accuracy model), yielding 630,476 raw reads (N50 = 2,819 bp).

Long reads were assembled and polished using Flye (v2.9.4-b1799) (4) and Medaka (v1.8.1) (5), yielding four contigs. CheckM (v1.2.2) (6) showed >99% completeness and 0.34% contamination. The automated circularization of the genome was performed by Flye and subsequently verified through visual inspection in Bandage (v0.8.1). The genome was not rotated to the *dnaA* start codon prior to annotation.

Coding regions were predicted by Pyrodigal (v3.12) (7) and filtered with AntiFam using default parameters (8), whereas tRNA and rRNA were identified using tRNAscan-SE 2.0 (9) and Infernal (10), respectively, yielding 1,939 unique CDS, 81 tRNA, and 18 rRNA genes (Table 1).

**TABLE 1:** Annotation and genome assembly statistics of *Chryseobacterium cupriresistens* Str01 isolated from a vegetable countertop in a public market in the Philippines^*a*^

Parameters	*Chryseobacterium cupriresistens* Str01
No. of raw reads	630,476
Genome size (bp)	5,077,165
No. of contigs	4
G + C content (%)	36.91
N50 (bp)	5,035,666
Genome coverage (×)	183
Genome completeness (%)	>99
Genome contamination (%)	0.34
Topology	Circular
Protein-coding genes	4,455
tRNA genes	81
rRNA genes	18
Pathogenicity^*b*^	Non-pathogenic
Genome accession number	JBRBXD000000000

^*a*^ The values given for genome completeness and contamination correspond to the polished genome.

^*b*^ The potential for pathogenicity is estimated using PathogenFinder2 v0.5.0.

Initial screening of the largest contig (~5 Mbp, 183×) via BLASTn (v2.15.0) (11) yielded alignment similarities of up to 98.55% with *C. indologenes* DSM16777 (12). However, average nucleotide identity (ANI) analysis via pyANI-plus (13) and *in silico* DNA-DNA hybridization using the TYGS server (14) revealed that relatedness to *C. indologenes* was far below the accepted species thresholds of 95% and 70%, respectively (ANIm = 85.20%, ANIb = 80.71%, and isDDH = 33.2%). Instead, the isolate shared greatest relatedness with *C. cupriresistens* EZn1T (ANIm = 95.48%*,* ANIb = 94.59%, and isDDH = 80.5%), a novel species isolated from heavy metal-contaminated soil in Mexico (15, 16). Consistent with the copper-resistant EZn1^T^, Str01 harbors genes for heavy-metal resistance.

Comprehensive annotation using ARMFinderPlus (v3.12.8) (17) and CARD-RGI (v.6.0.5) (18) revealed several virulence-associated genes (e.g., T6SS- and T9SS-associated genes, *vgr*, *sodA*, *luxR*, and *frnE*) and antimicrobial resistance genes (e.g., *adeF*, *aadS*, *blaCHM*, *blaCGA*, and *blaCGB*). Notably, while these genes were present, PathogenFinder 2.0 (v0.5.0) classified Str01 as non-pathogenic (19).

## Data Availability

This genome project is indexed at GenBank under BioProject accession number PRJNA1330406. The genome sequence of *C. cupriresistens* Str01 has been deposited in GenBank and Sequence Read Archive under accession numbers JBRBXD010000001.1 and SRR36705880, respectively. The version described in this paper is version JBRBXD010000001.1.
